# Comparing derivatization reagents for quantitative LC–MS/MS analysis of a variety of vitamin D metabolites

**DOI:** 10.1007/s00216-023-04753-0

**Published:** 2023-05-23

**Authors:** Anastasia Alexandridou, Pascal Schorr, Dietrich A. Volmer

**Affiliations:** Bioanalytical Chemistry, Department of Chemistry, Humboldt University Berlin, Brook-Taylor-Str. 2, 12489 Berlin, Germany

**Keywords:** Vitamin D_3_ metabolites, 25-Hydroxyvitamin D_3_, LC–MS/MS, Electrospray, Chemical derivatization, Epimers

## Abstract

The present study systematically compares the sensitivity and selectivity of the analysis of multiple vitamin D metabolites after chemical derivatization using different reagents for liquid chromatography-tandem mass spectrometry (LC–MS/MS). Generally, chemical derivatization is applied to vitamin D metabolites to increase the ionization efficiency, which is particularly important for very low abundant metabolites. Derivatization can also improve the selectivity of the LC separation. A wide variety of derivatization reagents has been reported in recent years, but information on their relative performance and applicability to different vitamin D metabolites is, unfortunately, not available in the literature. To fill this gap, we investigated vitamin D_3_, 3β-25-hydroxyvitamin D_3_ (3β-25(OH)D_3_), 3α-25-hydroxyvitamin D_3_ (3α-25(OH)D_3_), 1,25-dihydroxyvitamin D_3_ (1,25(OH)_2_D_3_), and 24,25-dihydroxyvitamin D_3_ (24,25(OH)_2_D_3_) and compared response factors and selectivity after derivatizing with several important reagents, including four dienophile reagents (4-phenyl-1,2,4-triazoline-3,5-dione (PTAD), 4-[2-(6,7-dimethoxy-4-methyl-3-oxo-3,4-dihydroquinoxalinyl)ethyl]-1,2,4-triazoline-3,5-dione (DMEQ-TAD), Amplifex, 2-nitrosopyridine (PyrNO)) as well as two reagents targeting hydroxyl groups: isonicotinoyl chloride (INC) and 2-fluoro-1-methylpyridinium-p-toluenesulfonate (FMP-TS). In addition, a combination of dienophiles and hydroxyl group reagents was examined. For LC separations, reversed-phase C-18 and mixed-mode pentafluorophenyl HPLC columns using different compositions of the mobile phase were compared. With respect to detection sensitivity, the optimum derivatization reagent for the profiling of multiple metabolites was Amplifex. Nevertheless, FMP-TS, INC, PTAD, or PTAD combined with an acetylation reaction showed very good performance for selected metabolites. These reagent combinations provided signal enhancements on the order of 3- to 295-fold depending on the compound. Chromatographic separation of the dihydroxylated vitamin D_3_ species was readily achieved using any of the derivatization reactions, while for 25(OH)D_3_ epimers, only PyrNO, FMP, INC, and PTAD combined with acetylation enabled complete separation. In conclusion, we believe this study can serve as a useful reference for vitamin D laboratories, to help analytical and clinical scientists decide which derivatization reagent to choose for their application.

**Supplementary Information:**

The online version contains supplementary material available at 10.1007/s00216-023-04753-0.

## Introduction

Liquid chromatography-tandem mass spectrometry (LC–MS/MS) is firmly established today as the gold standard technique for determining the vitamin D status (= concentration of 25-hydroxyvitamin D (25(OH)D) metabolites in human serum/plasma) of individuals [1–4]. While the detection sensitivity for this secosteroid is higher than for many other sterols and oxysterols, and thus sufficiently high for quantification of typical physiological levels of 25(OH)D, the detection of very low concentration levels, e.g., those seen in highly deficient individuals, is more challenging using typical MS assays. In addition, lower abundant metabolites such as the 3α-25(OH)D_3_ epimer, 24,25-dihydroxyvitamin D (24,25(OH)_2_D), or 1,25-dihydroxyvitamin D (1,25(OH)_2_D) are increasingly difficult to analyze, as their concentration levels are often only in the picograms per milliliter range, in particular levels of 1,25(OH)_2_D [5].

One option to enhance the sensitivity is to preconcentrate the analytes of interest [6]. Unfortunately, this is often challenging because of matrix interferences or sample size restrictions. Alternatively, selective chemical derivatization can be performed to increase response factors of the analytes.

Chemical derivatization of vitamin D compounds has been shown to increase ionization efficiency under ESI conditions, shift the masses of the analytes to higher *m/z* values with less isobaric noise from interferences, and provide more specific fragmentation patterns under MS/MS conditions [7]. Potential targets for chemical derivatization of vitamin D compounds are the highly specific *cis*-diene moiety at C-10/C-19 and C-5/C-6 as well as the less specific hydroxyl groups.

In this study, we investigated a wide range of commercially available derivatization reagents and compared analytical figures of merit such as sensitivity, chromatographic selectivity and resolution, derivatization time, ease of implementation, and analysis cost: 4-phenyl-1,2,4-triazoline-3,5-dione (PTAD), Amplifex, and 4-[2-(6,7-dimethoxy-4-methyl-3-oxo-3,4-dihydroquinoxalinyl)ethyl]-1,2,4-triazoline-3,5-dione (DMEQ-TAD), which have been widely used [8–21] for vitamin D metabolites. Isonicotinoyl chloride (INC) and 2-nitrosopyridine (PyrNO) have also been reported for vitamin D_3_ measurements [22–24]. In addition, 2-fluoro-1-methylpyridinium-p-toluenesulfonate (FMP-TS) was included in our investigation as reagent, to target the hydroxyl groups, which to our knowledge has not been used for vitamin D analysis before. We also included a double PTAD-acetylation one-pot reaction, which provides significantly enhanced sensitivity and improved chromatographic separation abilities.

In the present work, we focused on two primary objectives for conducting derivatization reactions for subsequent LC–MS/MS analysis: increased response factors for the analytes of interest and improved separation abilities for isomers and epimers during chromatography. As target metabolites, we chose vitamin D_3_, 3β-25(OH)D_3_, 3α-25(OH)D_3_, 1,25(OH)_2_D_3_, and 24,25(OH)_2_D_3_. The comparisons were conducted in electrospray ionization (ESI) positive ion mode using multiple reaction monitoring (MRM) on a triple quadrupole mass spectrometer. The chromatographic separation properties were investigated using four different chromatographic methods.

## Materials and methods

### Chemicals and materials

Standards of 3β-25(OH)D_3_ and 1,25(OH)_2_D_3_ were purchased from Cayman Chemical (Ann Arbor, MI, USA); vitamin D_3_ and (24R)-24,25(OH)_2_D_3_ from Toronto Research Chemicals (Toronto, ON, Canada); 3α-25(OH)D_3_, 4-phenyl-1,2,4-triazoline-3,5-dione, 2-fluoro-1-methylpyridinium-p-toluenesulfonate, isonicotinoyl chloride hydrochloride (95%), nicotinoyl chloride hydrochloride (97%), acetic anhydride (≥ 99%), pyridine (anhydrous, 99.8%), acetonitrile (anhydrous, 99.8%), and 4-dimethylaminopyridine (99%) from Sigma-Aldrich (Steinheim, Germany); acetic acid and triethylamine (≥ 99.5%) from Carl Roth (Karlsruhe, Germany); 4-[2-(6,7-dimethoxy-4-methyl-3-oxo-3,4-dihydroquinoxalinyl)ethyl]-1,2,4-triazoline-3,5-dione from Enzo Life Sciences (NY, USA); 2-nitrosopyridine from MedChemExpress (Monmouth Junction, NJ, USA); Amplifex Diene Reagent Kit from Sciex (Darmstadt, Germany); and 3β-25(ΟΗ)D_3_-[26,26,26,27,27,27-d6] monohydrate from IsoSciences (Ambler, PA, USA). UHPLC-MS-grade acetonitrile and methanol were obtained from Chemsolute (Th. Geyer, Renningen, Germany) and formic acid (97%) from Alfa Aesar (Karlsruhe, Germany). Organic-free water was generated by a Millipore (Bedford, MA, USA) Direct-Q8 purification system. Human vitamin D_3_-free serum (VD-DDC Mass Spect Gold® serum) was purchased from Sigma-Aldrich.

### Preparation of standard solutions and serum samples

Solid analytical standards of the investigated vitamin D_3_ compounds were dissolved in methanol to obtain 1 mg mL^−1^ stock solutions, which were stored at − 20 °C. Two groups of working solutions for the chromatographic investigations were prepared in methanol/water 90/10 (v/v). Group A included 3β-25(OH)D_3_ at 20 ng mL^−1^, vitamin D_3_ at 30 ng mL^−1^, and 1,25(OH)_2_D_3_ at 10 ng mL^−1^; group B included 3α-25(OH)D_3_ at 5 ng mL^−1^ and (24R)-24,25(OH)_2_D_3_ at 10 ng mL^−1^. Blank working solutions consisted of methanol/water 90/10 (v/v).

Human vitamin D_3_-free serum was spiked with vitamin D standard solutions to give final concentrations of 3β-25(OH)D_3_, vitamin D_3_, 1,25(OH)_2_D_3_, 3α-25(OH)D_3_, and (24R)-24,25(OH)_2_D_3_ of 20 ng mL^−1^, 30 ng mL^−1^, 10 ng mL^−1^, 5 ng mL^−1^, and 10 ng mL^−1^, respectively. The spiked vitamin D-free serum samples were divided into two groups. Group A included 3β-25(OH)D_3_, vitamin D_3_, and 1,25(OH)_2_D_3_, and group B included 3α-25(OH)D_3_ and (24R)-24,25(OH)_2_D_3_. This two-group split ensured that the retention times of the epimers or isobars and the peak areas were readily determined under the applied chromatographic conditions.

The sample preparation protocol applied was adapted from our previous protocol [25]. Briefly, 250 μL of acetonitrile was added to 100 μL of serum to precipitate proteins and dissociate compounds from binding proteins, followed by 1 min of vortexing and 15 min of centrifugation at 10.000 rpm. The supernatant was transferred to a new vial, and the sample was evaporated to dryness using a Concentrator plus/Vacufuge® plus (Eppendorf, Hamburg, Germany). Subsequently, two-step liquid–liquid extraction (LLE) was performed. For LLE, 100 μL of water and 200 μL of ethyl acetate were added to the dry residue followed by 30 s of vortexing and 5 min of centrifugation at 10.000 rpm. The upper organic phase was transferred to a new vial, and the remaining water phase was re-extracted by adding 200 μL of ethyl acetate. The two resulting organic fractions were combined, and 380 μL of the 400 μL total extract volume was evaporated to dryness prior to derivatization, to ensure reproducible liquid transfer.

For the comparison, it was important that the chosen sample preparation procedure did not influence the results between different derivatization reagents. For this reason, LLE was performed for 50 spiked serum samples and the two organic phases from every LLE experiment were combined to approximately 20 mL total volume. Subsequently, in fresh vials, 380 μL of the combined volume was transferred and evaporated to dryness. The residues were then made to undergo derivatization with the respective reagent. The internal standard was 25(OH)D_3_-d6, which was added at 10 ng mL^−1^ after every derivatization reaction at the reconstituting stage.

For every investigated derivatization reagent, the extraction protocol was applied to 100 μL of the working solution: two samples belonging to group A and two samples belonging to group B. Every working solution sample from each group was measured in triplicate. Three spiked serum samples from group A and three spiked serum samples from group B were prepared for every derivatization reagent. Every sample was measured in duplicate.

### Derivatization procedures

#### PTAD

PTAD was accurately weighed and dissolved in dry pure acetonitrile for a final concentration of 0.5 mg mL^−1^; 50 μL of this solution was added to the dried sample and the mixture was kept at room temperature for 60 min in the dark. After addition of 50 μL of MeOH to decompose any excess reagent, the sample was vortexed and the solvent was evaporated. The sample was reconstituted in 100 μL of a mixture of MeOH:H_2_O 90/10 (v/v) and transferred to a LC vial.

##### PTAD and acetylation

For the one-pot reaction, 100 μL of serum was dried and PTAD solution (0.50 mg mL^−1^, 33.3 μL) containing 2% of acetic acid was added to the dried sample; the mixture was kept at room temperature for 60 min in the dark. Subsequently, 33.3 μL of a mixture of pyridine:acetic anhydride 67/33 (v/v) including 2 mg mL^−1^ of DMAP was added and the reaction performed at room temperature for 60 min in the dark. Importantly, the mixture of pyridine:acetic anhydride 67/33 (v/v) should be prepared directly before use. After the addition of 50 μL of methanol, the solvent was evaporated and the sample was reconstituted in 100 μL of a mixture of MeOH:H_2_O 90/10 (v/v) prior to analysis.

##### Amplifex diene reagent

Amplifex diene reagent solution was prepared as described by the manufacturer. To the dried sample, 50 μL of Amplifex diene reagent was added and the mixture was vortexed for 30 s. The reaction was conducted at ambient temperature for 30 min. After adding 50 μL of methanol, the solvent was evaporated and the sample was reconstituted in 100 μL of a mixture of MeOH:H_2_O 90/10 (v/v) prior to injection.

#### FMP-TS

The derivatization reaction was adopted from Faqehi et al., who used FMP-TS to derivatize estrogens [26]. To the dried sample, 50 μL of a freshly prepared solution of FMP-TS (5 mg mL^−1^) in dry acetonitrile containing 1% triethylamine was added and the mixture was vortexed for 15 s. After incubation for 15 min at 40 °C, the reaction was quenched by adding 50 μL of methanol. The solvent was evaporated, and the sample was reconstituted using 100 μL of a mixture of MeOH:H_2_O 90/10 (/v/v) before being transferred to an LC vial.

##### Isonicotinoyl chloride

A saturated isonicotinoyl chloride solution (5 mg mL^−1^) was prepared in acetonitrile. After ultrasonication (5 min) and centrifugation (5 min), the supernatant was used for derivatization. DMAP solution (10 mg mL^−1^) was prepared in acetonitrile. The dried sample was dissolved in 100 μL of acetonitrile, and 10 μL of the derivatization reagent was added as well as 10 μL of DMAP solution. The mixture was vortexed for 10 s and subsequently dried. Before analysis, the sample was reconstituted using 100 μL of a mixture of MeOH:H_2_O 90/10 (v/v).

##### 2-Nitrosopyridine

A methanolic solution of PyrNO (2.5 mM) was prepared according to Wan et al. [24] by weighting 0.27 mg of PyrNO and dissolving it in 1 mL of methanol; 40 μL of the derivatization reagent was added to the dried samples, and the reaction took place at 70 °C for 60 min. The samples were left to cool down, the solvent was evaporated to dryness, and reconstitution using 100 μL of a mixture of MeOH:H_2_O 90:10 (v/v) was conducted as the final step prior to analysis.

#### DMEQ-TAD

The derivatization reaction was conducted as described by Kaufmann et al. [15]. A solution of DMEQ-TAD was prepared in ethyl acetate at 0.1 mg mL^−1^. To the dried sample, 25 μL of DMEQ-TAD solution was added. The sample was incubated at ambient temperature for 30 min. Subsequently, a second addition of 25 μL of DMEQ-TAD solution was performed, followed by 60 min of incubation. To quench the reaction, 50 μL of methanol was added and the solvent was evaporated to dryness. The sample was reconstituted using 100 μL of a mixture of MeOH:H_2_O 90/10 (v/v) and transferred to a LC vial.

### Liquid chromatography-tandem mass spectrometry

For LC–MS/MS experiments, 5 μL of a sample was injected using a 1290 Infinity II LC system (Agilent, Wilmington, DE, USA). Two different stationary phases and two different mobile phases were investigated for each derivatization reagent. The columns were Phenomenex (Torrance, CA, USA), Kinetex 2.6 µm C-18 100 Å (100 × 2.1 mm), and Kinetex 2.6 µm F5 100 Å (100 × 2.1 mm). The mobile phases were (A1) water (+ 0.1% formic acid) and (B1) methanol (+ 0.1% formic acid) or (A2) water (+ 0.1% formic acid) and (B2) acetonitrile (+ 0.1% formic acid). The gradient was linearly increased from 50 to 100% B within 15 min and then held for 2 min at 100% B, before returning to the initial conditions for re-equilibration for 3 min. The flow rate was 0.4 mL/min, and the temperature was set to 30 °C for all experiments.

The UHPLC system was coupled to a Sciex (Concord, ON, Canada) QTRAP 6500^+^ quadrupole–quadrupole-linear ion trap mass spectrometer equipped with an IonDrive Turbo-V ESI source. ESI was conducted in positive ion mode under MRM conditions. Ion source parameters were as follows: IonSpray voltage: 5500 V; source temperature: 300 °C; curtain gas: 35 psi; ion source gas 1 (nebulizer gas): 30 psi; ion source gas 2 (heating gas): 30 psi; and the collision gas was set to medium. For the MRM experiments, declustering potential (DP), entrance potential (EP), collision energy (CE), and collision cell exit potential (CXP) were optimized for each vitamin D metabolite derivative separately and the obtained settings are summarized in Table S1 (Supplementary information). The dwell times (ms) were adjusted to obtain a minimum of 12 data points across the chromatographic peaks.

Data acquisition was performed using Analyst software (Sciex) version 1.7 and MultiQuant (Sciex) version 3.0.3.

## Results and discussion

In this study, we sought to find the optimum derivatization reagent for the determination of multiple low abundant vitamin D metabolites in serum, selected from a wide variety of commercially available reagents. We defined the optimum performance with respect to sensitivity of analysis as well as the chromatographic separation ability for vitamin D isomers and isobars. We purposely chose readily available, commercial derivatization reagents to allow for simple implementation in vitamin D assays from serum/plasma samples. Specifically, the different reagents were compared for LC–MS/MS analysis of five different vitamin D_3_ metabolites. In addition, the separation abilities of two common stationary phases under different mobile phase conditions were investigated for the derivatized vitamin D compounds.

PTAD, Amplifex, DMEQ-TAD, and PyrNO perform a Diels–Alder reaction, where PTAD and Amplifex are firmly established reagents and frequently implemented for metabolite profiling. FMP-TS and INC react with the hydroxyl groups of the analytes and are thus far less specific for vitamin D_3_ metabolite analysis. To our knowledge, FMP-TS has not been used for vitamin D_3_ metabolite measurements. Finally, the combination of Diels–Alder reaction using PTAD and acetylation of the hydroxyl groups was investigated in comparison to the other reagents.

We expected Amplifex and FMP-TS to enhance detection sensitivity the most under ESI conditions, based on the permanently charged moiety in the reagent core structures. For the chromatographic separation of the epimers and isomers, we were looking for improved selectivity after derivatization, as some of the underivatized metabolites are sometimes difficult to fully separate under commonly applied chromatographic conditions.

### MS/MS dissociation behavior of the derivatization products

In this section, we briefly summarize the observed fragmentation patterns for the products of the investigated derivatization reactions. A more detailed description of the precursor ions and dissociation behavior is summarized in Scheme 1 (Supplementary information).

**Fig. 1:**
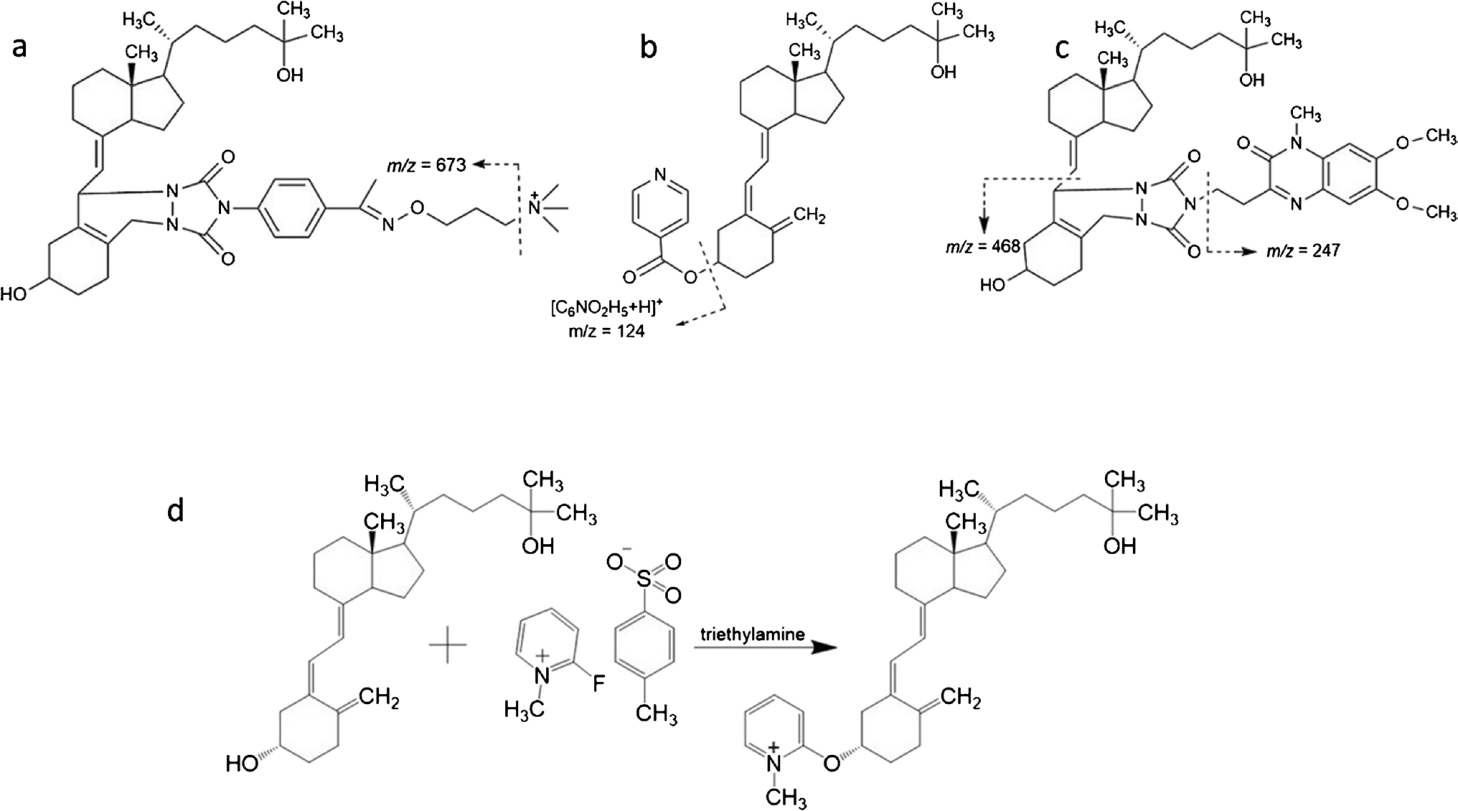
Fragmentation patterns of the derivatization products of 25(OH)D_3_: (**a**) Amplifex; (**b**) INC; (**c**) DMEQ-TAD; (**d**) FMP-TS in the presence of triethylamine

Amplifex reacts as dienophile in a hetero-Diels–Alder reaction with vitamin D_3_ metabolites. The positively charged quaternary ammonium group is the common leaving group during fragmentation for all analytes at *m/z* 59 (Fig. 1(a)).

FMP-TS reacts with hydroxyl groups, producing a permanently charged pyridinium moiety through a nucleophilic substitution. For vitamin D_3_ species, all non-tertiary hydroxyl groups can potentially react with the reagent. Figure 1(d) shows the reaction of FMP-TS with 3β-25(OH)D_3_, which can be performed with the hydroxyl group at C-3; it does not take place at C-25 due to stereochemical hindrances.

INC performs an acylation reaction at the hydroxyl groups to give esters [27]. Usually, pyridine or a pyridine derivative is utilized to neutralize the hydrochloric acid, which is a side product of the reaction. More details on the precursor ions of the investigated metabolites are given in Scheme S1 (Supplementary information). The common product ion for all analytes was at *m/z* 124, corresponding to protonated isonicotinic acid [C_6_NO_2_H_5_ + H]^+^ (Fig. 1(b)).

DMEQ-TAD is a Cookson-type reagent such as PTAD or Amplifex. The major product ion after fragmentation is seen at *m/z* 247 for all derivatized analytes, from cleavage of the bond between DMEQ and TAD. In this study, we selected *m/z* 468 for MRM, which is an A-ring-derived DMEQ-TAD fragment (Fig. 1(c)). This product ion offered lower background noise and greater specificity than the base peak in the CID spectrum, which was also shown by Kaufmann et al. [15].

### Detection sensitivity comparison of the investigated derivatization reagents

The seven derivatization reagents were systematically compared in terms of enhancement of the ionization efficiency. The response factor enhancement was expressed as a relative peak area, that is, the ratio of the peak area of the derivatized to the non-derivatized analyte (Table 1). MRM transitions were selected for interference-free chromatographic traces in the extracted ion chromatograms of blank serum samples, to obtain maximum signal intensities.

**Table 1:** Relative peak area ratios for the five investigated metabolites

Spiked serum samples (peak area derivatized/peak area non-derivatized)				
Derivative	C-18/H_2_O-MeOH	C-18/H_2_O-ACN	F5/H_2_O-MeOH	F5/H_2_O-ACN
1,25(OH)_2_D_3_-PTAD	24	10	24	13
24,25(OH)_2_D_3_-PTAD	4	15	4	7
3β-25(OH)D_3_-PTAD	3	4	4	26
3α-25(OH)D_3_-PTAD	4	1	15	24
D_3_-PTAD	19	25	7	12
1,25(OH)_2_D_3_-PTAD + Ac	3	3	3	4
24,25(OH)_2_D_3_-PTAD + Ac	1	7	1	4
3β-25(OH)D_3_-PTAD + Ac	1	2	2	8
3α-25(OH)D_3_-PTAD + Ac	1	1	2	4
D_3_-PTAD + Ac	3	7	4	4
1,25(OH)_2_D_3_-Amplifex	349	81	329	211
24,25(OH)_2_D_3_-Amplifex	27	14	28	28
3β-25(OH)D_3_-Amplifex	47	39	55	141
3α-25(OH)D_3_- Amplifex	47	19	167	127
D_3_-Amplifex	271	331	112	-
1,25(OH)_2_D_3_-FMP	5	3	8	3
24,25(OH)_2_D_3_-FMP	5	2	9	12
3β-25(OH)D_3_-FMP	13	10	10	33
3α-25(OH)D_3_-FMP	15	6	34	30
D_3_-FMP	262	245	295	285
1,25(OH)_2_D_3_-INC	18	9	11	13
24,25(OH)_2_D_3_-INC	6	4	6	18
3β-25(OH)D_3_-INC	6	13	6	36
3α-25(OH)D_3_-INC	16	18	33	45
D_3_-INC	4	-	3	9
1,25(OH)_2_D_3_-PyrNO	2	8	7	10
24,25(OH)_2_D_3_-PyrNO	1	11	2	3
3β-25(OH)D_3_-PyrNO	2	3	2	13
3α-25(OH)D_3_-PyrNO	1	1	5	14
D_3_-PyrNO	16	7	2	9
1,25(OH)_2_D_3_-DMEQ-TAD	13	14	14	6
24,25(OH)_2_D_3_-DMEQ-TAD	1	7	1	3
3β-25(OH)D_3_-DMEQ-TAD	1	7	1	13
3α-25(OH)D_3_-DMEQ-TAD	1	1	2	12
D_3_-DMEQ-TAD	28	30	2	16

Chromatographic peak areas were calculated from the extracted ion traces of the MRM experiments using the instrument quantification software (see “Materials and methods”)

As is evident from Table 1, the optimum derivatization reagent for all investigated metabolites (except for the native vitamin D_3_) was Amplifex, which enhanced responses 14–331-fold. This strong enhancement was likely due to the preformed positive charge from the Amplifex reagent (Fig. 1(a)), which strongly improved ESI response. Similarly, FMP-TS products exhibited strongly enhanced responses, which was again due to the permanent positive charge of the products. Derivatization with FMP-TS also provided a strong increase of the response factor for native vitamin D_3_ (Table 1).

When comparing the dihydroxylated species, we noticed that Amplifex increased the response of 1,25(OH)_2_D_3_ almost tenfold stronger than 24,25(OH)_2_D_3_ (Table 2). This was observed regardless of the mobile phase used and thus cannot be explained by the different separations for the two stereoisomers (6S and 6R) of 24,25(OH)_2_D_3_ and 1,25(OH)_2_D_3_, depending on the mobile phase. We attribute this difference to different ion suppression effects for the various species. Interestingly, the opposite trend was seen for 25(OH)D_3_ epimers, where higher detection sensitivity was obtained when H_2_O-ACN was used as mobile phase (Table 1).

**Table 2:** Calculated resolution values for the investigated dihydroxylated vitamin D_3_ metabolites

1,25(OH)_2_D_3_ and 24,25(OH)_2_D_3_—resolution (*R*_*s*_)				
Compounds	Chromatographic conditions (column/mobile phase)			
	C-18/H_2_O-MeOH	C-18/H_2_O-ACN	F5/H_2_O-MeOH	F5/H_2_O-ACN
1,25(OH)_2_D_3_ and 24,25(OH)_2_D_3_	2.1	0.8	5.3	0.3
1,25(OH)_2_D_3_-PTAD and 24,25(OH)_2_D_3_-PTAD	4.6	1.5	3.7	0.6
1,25(OH)_2_D_3_-PTAD-Ac and 24,25(OH)_2_D_3_-PTAD-Ac	1.9	3.2	0.8	2.1
1,25(OH)_2_D_3_-Amplifex and 24,25(OH)_2_D_3_-Amplifex	3.7	0.4	2.7	0.5
1,25(OH)_2_D_3_-FMP-TS and 24,25(OH)_2_D_3_-FMP-TS	5.3	1.7	4.4	1.5
1,25(OH)_2_D_3_-INC and 24,25(OH)_2_D_3_-INC	7.9	5.4	1.6	2.4
1,25(OH)_2_D_3_-PyrNO and 24,25(OH)_2_D_3_-PyrNO	5.1	0.0	3.8	0.3
1,25(OH)_2_D_3_-DMEQ-TAD and 24,25(OH)_2_D_3_-DMEQ-TAD	3.1	3.6	10.9 (Fig. 3)	0.1

PTAD products generally exhibited the same behavior, with enhancement factors in the range between 3 and 25. Usually, the 1,25(OH)_2_D_3_–PTAD products coeluted in contrast to the reaction products of 24,25(OH)_2_D_3_. For the PTAD products of 25(OH)D_3_ epimers, higher sensitivity was shown for the H_2_O-ACN mobile phase. Higashi et al. observed a 100-fold sensitivity increase after PTAD derivatization of 25(OH)D_3_ in saliva [11]. The authors pointed out the importance of the methylamine addition to the mobile phase. Aronov et al. report a 100-fold increase of the analytical signal for PTAD-derivatized 1,25(OH)_2_D_3_ [13], which was similar to Xue et al., who measured 25(OH)D_3_ and 24,25(OH)_2_D_3_ in rat serum and brain tissue and increased their assay sensitivity by 100-fold after derivatizing with PTAD [28]. Both Aronov et al. and Xue et al. used formic acid as a modifier. The enhancement factors reported in our study were slightly smaller, but the differences are likely due to the different additives to the mobile phase and the different sample matrices. Importantly, signal enhancement was calculated differently between the studies, which likely also led to different absolute numbers.

Bonnet et al. analyzed adipose tissue for 25(OH)D_3_ and 1,25(OH)_2_D_3_ after Amplifex and PTAD derivatization [8]. The authors obtained better performance for Amplifex than PTAD. Hedman et al. compared Amplifex to PTAD for 1,25(OH)_2_D in serum samples [29]. The Amplifex method gave tenfold higher S/N than PTAD. The data from our study are in general agreement with these studies (Table 2).

PTAD-Ac products exhibited 2–8 times lower detection sensitivity than PTAD products, which may be due to the different chromatographic separation of the stereoisomers. Nevertheless, PTAD-Ac derivatization offers an improved chromatographic separation of the 25(OH)D_3_ epimers (see the following section for more details). Higashi et al. introduced a second step of derivatization of the C-3 hydroxyl group of 25(OH)D_3_ to separate the two 25(OH)D_3_ epimers [30]. The procedure increased the response 40-fold in comparison to the non-derivatized analyte. Our results exhibited an eightfold increase for 25(OH)D_3_. Higashi et al. used ammonium formate to promote a specific methylamine-adduct during ESI [30], while our method only used formic acid.

FMP and PyrNO products of the mono-hydroxyl and di-hydroxyl species exhibited very consistent behavior: there was almost no difference between 3α-25(OH)D_3_ and 3β-25(OH)D_3_, and between 1,25(OH)_2_D_3_ and 24,25(OH)_2_D_3_ products under all given chromatographic conditions. PyrNO derivatives performed better on a PFP column in combination with H_2_O-ACN, enhancing detection sensitivity tenfold as compared to the non-derivatized metabolites, except for 24,25(OH)_2_D_3_. Wan et al. reported a fivefold increase in signal intensity for 1,25(OH)_2_D_3_-PyrNO as compared to 1,25(OH)_2_D_3_-PTAD under optimized conditions for each reagent [24]. Helmeczi et al. observed a tenfold enhancement of ionization efficiency for 25(OH)D_3_-PyrNO as compared to the non-derivatized 25(OH)D_3_[22], similar to our findings.

To our knowledge, this study reports derivatization of vitamin D_3_ metabolites using FMP-TS for the first time, while derivatization of estrogens with FMP-TS has been reported before [26, 31]. The estrogens’ FMP signal intensity increased 3–31-fold as compared to the non-derivatized species [31], which is similar to our vitamin D data, where sensitivity was increased 2–34-fold for all investigated vitamin D metabolites, except for vitamin D_3_, which showed even greater increase (272 ×). Additives such as formic acid in the mobile phase and the final reconstitution of the samples before measurement, however, can play a major role in ion suppression and the final signal intensities [31].

INC products of 3α-25(OH)D_3_ and 1,25(OH)_2_D_3_ showed higher signal enhancement than 3β-25(OH)D_3_ and 24,25(OH)_2_D_3_, with signals 3–45 times higher than the underivatized molecules, depending on the mobile phase and column used. In comparison, Le et al. reported that S/N values improved 200–1000-fold using INC derivatization, as compared to non-derivatized analytes [23].

DMEQ-TAD is a sensitive fluorophore and has thus previously been used for vitamin D_3_ metabolite measurements in fluorometric methods [16, 17, 32, 33]. DMEQ-TAD demonstrated excellent behavior for 1,25(OH)_2_D_3_ and vitamin D_3_ for all mobile phases (Table 2) and was equally suited for the other vitamin D metabolites on the PFP column and H_2_O/ACN mobile phase (Table 1). Higashi et al. reported DMEQ-TAD for 25(OH)D_2_ and 25(OH)D_3_ using LC-APCI-MS/MS with 15-fold improved sensitivity [18].

In conclusion, significant increases for the response factors were seen with most of the investigated reagents, but large individual differences were observed. The variations seen for response factor improvements in our study and compared to literature data are likely mostly the result of differences in the chromatographic methods, that is, differences of stationary and mobile phase selectivity, column efficiency, additives, and ion suppression effects.

### Chromatographic separations of derivatization products

Four chromatographic methods were evaluated for the separation of the five investigated vitamin D metabolites after derivatization. We used C-18 and PFP stationary phases as well as two different mobile phases (acetonitrile/water and methanol/water gradients) for each column. The detailed experimental results of these experiments are summarized in Table S2 of the Supplementary information. The main goal of these separations was to evaluate the potential to separate the various epimers and isomers of vitamin D, that is, the ability to separate 3β-25(OH)D_3_ from 3α-25(OH)D_3_ (first critical pair) as well as 1,25(OH)_2_D_3_ from 24,25(OH)_2_D_3_ (second critical pair), with particular emphasis on the multiple products formed for each of the metabolites during the derivatization reactions. Importantly, the goal of this study was not the optimization of all the investigated metabolites; therefore, peak resolution (*R*_s_) was used only to evaluate the separation of the two critical pairs.

#### Number of products formed during derivatization

**Fig. 2:**
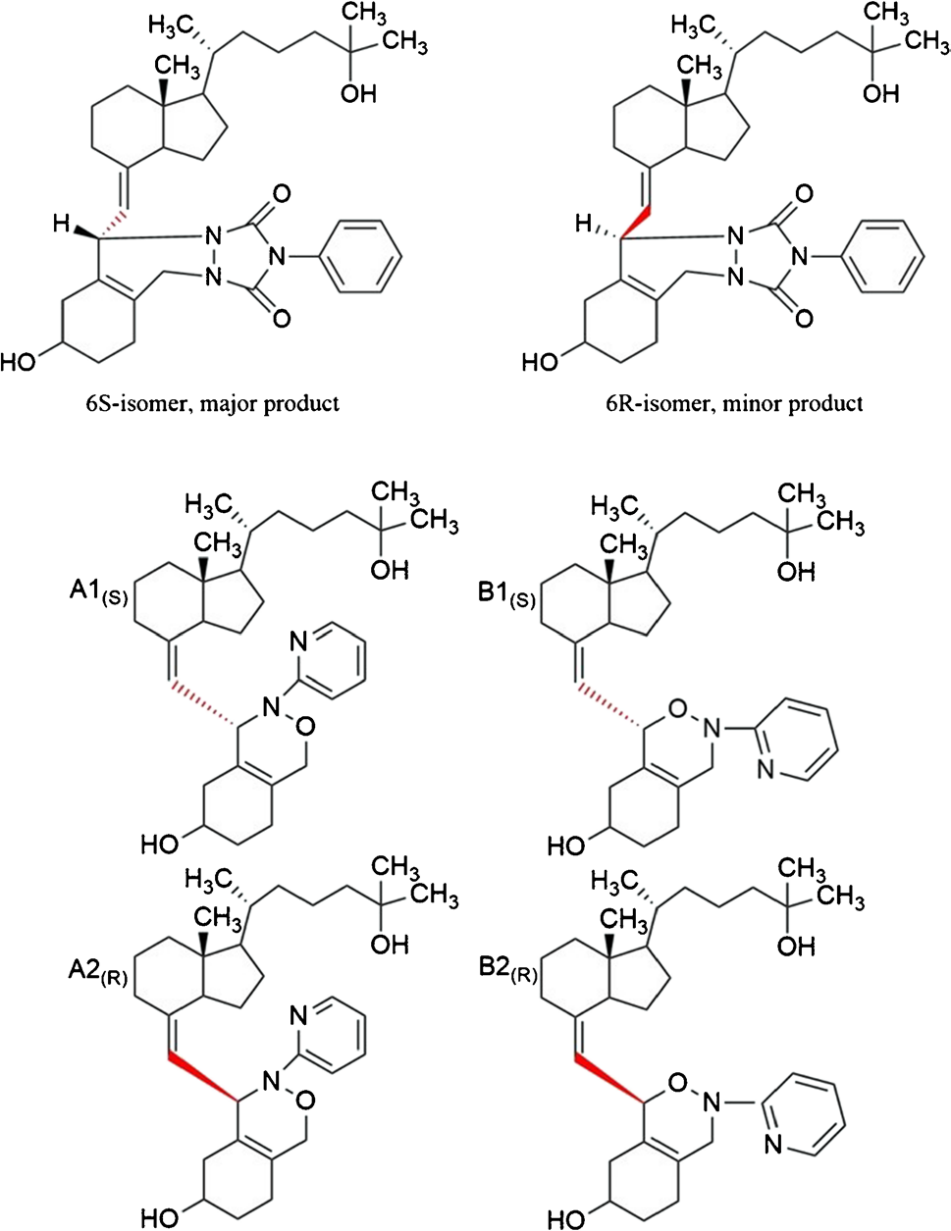
(Top) Diastereomers (6S and 6R) of the 25(OH)D_3_-PTAD product. (Bottom**)** Regioisomers (A and B) and diastereomers (A1_(S)_-A2_(R)_, B1_(S)_-B2_(R)_) of 25(OH)D_3_-PyrNO product

As discussed above, reagents attacking the *cis*-diene moiety of vitamin D compounds from the α- and β-sides of the molecule produce two stereoisomers (6R and 6S; Fig. 2, top) [12, 15, 18–20, 28, 34–36]. Specifically, for 25(OH)D_3_-PTAD, the ratio of 6S/6R is approximately 4:1 [11, 25], while for DMEQ-TAD, the 6S stereoisomer of the DMEQ-TAD product is the more abundant species [15, 18, 35]. Four isomers are produced in the reaction between vitamin D_3_ metabolites and PyrNO: two regioisomers, each of which is composed of two diastereomers (Fig. 2, bottom). As a result, four peaks were observed for each metabolite in our experiments, in contrast to Wan et al., who reported only two peaks [24]. Each pair of peaks produced the same ions after ESI, yet these ion pairs appeared in different ratios.

For PTAD-Ac products of 1,25(OH)_2_D_3_, 24,25(OH)_2_D_3_ and vitamin D_3_, two peaks were expected from the Diels–Alder reaction (6R and 6S). The total number of observed products, however, was four, corresponding to 25(OH)D_3_-PTAD-Ac epimers. This can be attributed to the chosen precursor ion (*m/z* 600), which corresponded to either [M_PTAD+Ac_ + H-H_2_O]^+^ or [M_PTAD+2×Ac_ + H-CH_3_COOH]^+^. During the reaction of PTAD products with pyridine:acetic anhydride at room temperature, two acetyl groups may be attached to 25(OH)D_3_, as a result of higher DMAP concentrations or elevated temperatures.

For all other derivatizations, a single product was seen in the chromatograms, except for the two peaks seen for 1,25(OH)_2_D_3_-INC, which can be readily explained by INC addition to the hydroxyl groups at C-1 and C-3, which both give the same precursor ion ([M_INC_ + H]^+^) at *m/z* 522.

#### Dihydroxylated species (1,25(OH)_2_D_3_ and 24,25(OH)_2_D_3_)

In this study, we used peak resolution (*R*_s_) to study the chromatographic effects of derivatization on the two critical pairs of vitamin D metabolites, 3β-25(OH)D_3_/3α-25(OH)D_3_ and 1,25(OH)_2_D_3_/24,25(OH)_2_D_3_. Both peak capacity and resolution were considered to assess the chromatographic quality of the gradient separations [37]. Peak capacity, however, is more useful to describe the separations of all investigated vitamin D metabolites or to compare different chromatographic systems. Moreover, higher peak capacity does not always guarantee improved separation of a specific peak pair [38], whereas resolution is a useful measure to describe the separation of a pair of analytes that are of a particular interest. We calculated resolution using Eq. (1), where *t*_p1_ and *t*_p2_ are the retention times of the analytes of the critical pair and *w*_p1_ and *w*_p2_ are the peak widths at the base of the peaks:

1 $${R}_{s}=\frac{2\times \left({t}_{\mathrm{p}1}-{t}_{\mathrm{p}2}\right)}{{w}_{\mathrm{p}1}+{w}_{\mathrm{p}2}}$$

To achieve baseline resolution, *R*_s_ should be ≥ 1.5 for symmetric peaks or ≥ 2.0 for peaks with some tailing [39].

Table 2 shows experimental resolution values for the dihydroxylated species 1,25(OH)_2_D_3_ and 24,25(OH)_2_D_3_ using the different investigated stationary and mobile phase conditions. In general, H_2_O/MeOH as mobile phase offered better resolving power than H_2_O/ACN for all the reagents, except for PTAD-Ac products. Equally, the C-18 stationary phase resolved the two derivatized analytes better than the fluorinated column, except for DMEQ-TAD products. In general, the products of most of the derivatization reagents were better resolved than their non-derivatized precursors. Importantly, even in those few cases where the *R*_s_ value was lower for the derivatization products than for the non-derivatized metabolites (e.g., PTAD-Ac or Amplifex), full baseline separation was achieved.

**Fig. 3:**
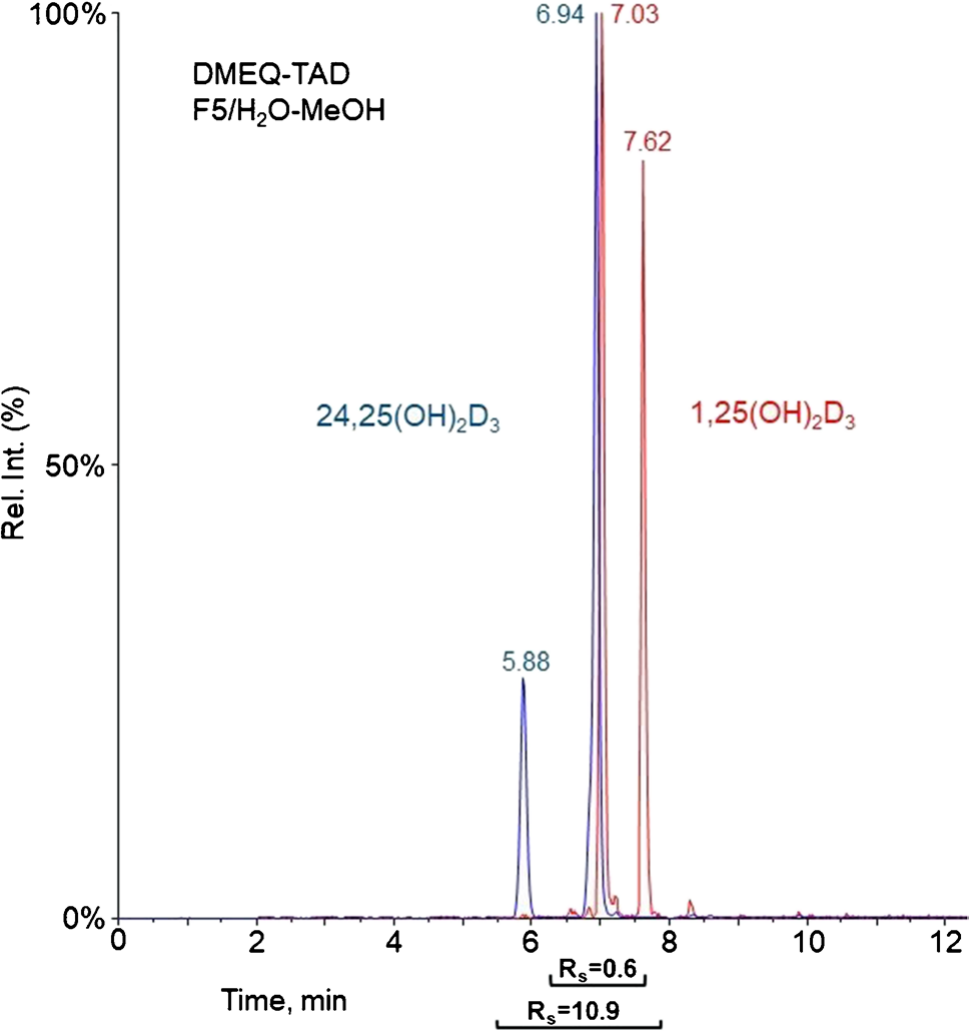
Chromatographic separation of DMEQ-TAD reaction products of 1,25(OH)_2_D_3_ (red) and 24,25(OH)_2_D_3_ (blue) on a perfluorinated column using H_2_O/MeOH (+ 0.1% formic acid) as mobile phase (square brackets below the chromatogram show the peak pairs with corresponding *R*_s_ value)

Specifically, for FMP-TS and INC products with 1.5 ≤ *R*_s_ ≤ 1.7, separation was readily achieved due to the narrow and symmetric peaks. The one-pot double PTAD-Ac derivatization reaction resulted in lower *R*_s_ for this peak pair compared to the single PTAD reaction. Under the investigated chromatographic conditions, PyrNO and DMEQ-TAD derivatization products were the most challenging to separate, because of coelution of several diastereomeric peaks. For PyrNO and DMEQ-TAD, some of the diastereomeric peaks of the analytes were separated, while others coeluted (Fig. 3).

#### C-3 epimers, 3β-25(OH)D_3_ and 3α-25(OH)D_3_

In comparison to 1,25(OH)_2_D_3_ and 24,25(OH)_2_D_3_, the epimeric 3β-25(OH)D_3_ and 3α-25(OH)D_3_ species were even more challenging to separate. Table 3 shows the experimental resolution values for the epimer peaks. We have previously demonstrated successful baseline separation of the C-3 epimers after Amplifex derivatization [40, 41].

**Table 3:** Calculated resolution values for mono-hydroxylated vitamin D_3_ metabolites

3β-25(OH)D_3_ and 3α-25(OH)D_3_—resolution (*R*_s_)				
Compounds	Chromatographic conditions (column/mobile phase)			
	C-18/H_2_O-MeOH	C-18/H_2_O-ACN	F5/H_2_O-MeOH	F5/H_2_O-ACN
3β-25(OH)D_3_ and 3α-25(OH)D_3_	0.0	0.0	1.6	0.5
3β-25(OH)D_3_-PTAD and 3α-25(OH)D_3_-PTAD	0.4	0.4	0.3	0.1
3β-25(OH)D_3_-PTAD-Ac and 3α-25(OH)D_3_-PTAD-Ac	2.9	3.1 (Fig. 4)	1.1	0.6
3β-25(OH)D_3_-Amplifex and 3α-25(OH)D_3_-Amplifex	0.0	0.2	0.0	0.0
3β-25(OH)D_3_-FMP-TS and 3α-25(OH)D_3_-FMP-TS	0.4	0.4	1.2	1.1
3β-25(OH)D_3_-INC and 3α-25(OH)D_3_-INC	0.0	0.5	2.3	1.5
3β-25(OH)D_3_-PyrNO and 3α-25(OH)D_3_-PyrNO	1.9	2.5	3.5 (Fig. 5)	0.3
3β-25(OH)D_3_-DMEQ-TAD and 3α-25(OH)D_3_-DMEQ-TAD	0.0	0.0	1.0	0.0

A general observation from the current study was that higher *R*_s_ values were always achieved for the C-3 epimers in comparison to the non-derivatized epimers, on both the C-18 and perfluorinated stationary phases, when H_2_O/ACN and H_2_O/MeOH were used as mobile phases. The data demonstrate that the perfluorinated column offered slightly better separation for most of the different derivatization products.

Baseline separation (*R*_s_ = 1.6) of the non-derivatized epimers was also observed on a perfluorinated column in combination with H_2_O/MeOH (+ 0.1% formic acid) as mobile phase. However, derivatization is crucial for improving the detection sensitivity of 3α-25(OH)D_3_, as this metabolite is usually present at very low concentration levels. Van den Ouweland et al. successfully measured the underivatized epimers on a pentafluorophenyl column [42] using LC–MS/MS, but applied the method to serum samples of newborns, where the 3α epimer is present at much higher concentrations than in adults [43].

**Fig. 4:**
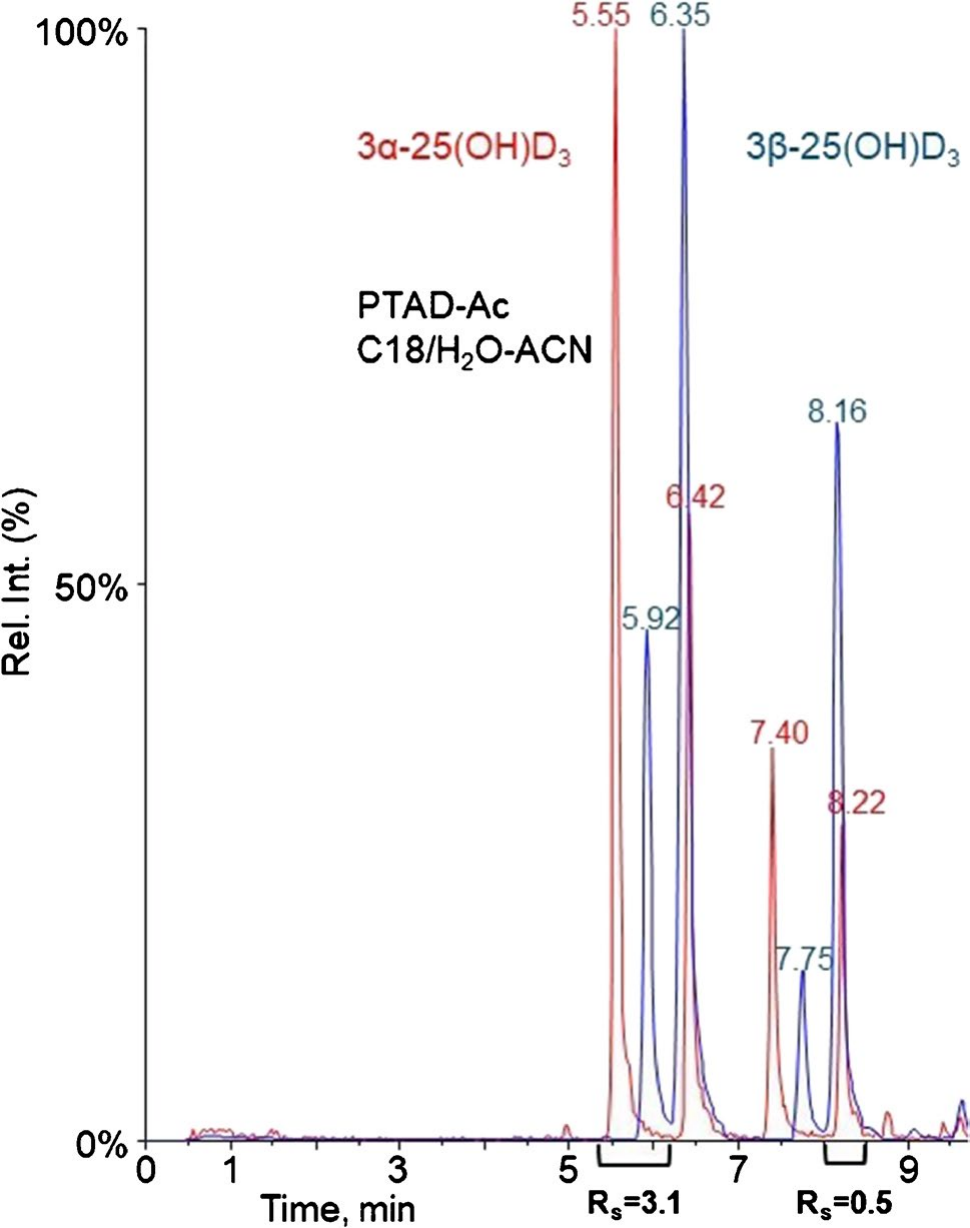
Chromatographic separation of products of 3α-25(OH)D_3_ (red) and 3β-25(OH)D_3_ (blue) after derivatization with PTAD-Ac using a C-18 column and H_2_O/ACN (+ 0.1% formic acid) as mobile phase (square brackets below the chromatogram show the peak pairs with corresponding *R*_s_ value)

**Fig. 5:**
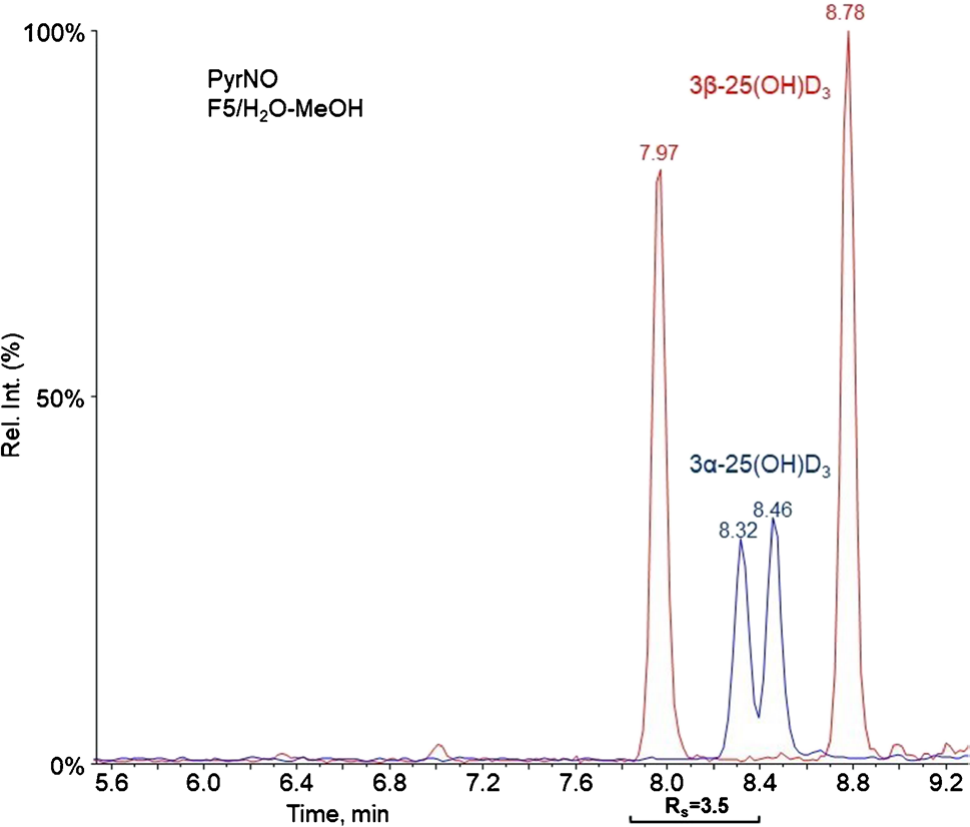
Chromatographic separation of products of 3α-25(OH)D_3_ (blue) and 3β-25(OH)D_3_ (red) after derivatization with PyrNO using a perfluorinated column and H_2_O/MeOH (+ 0.1% formic acid) as mobile phase (square brackets below the chromatogram show the peak pairs with corresponding *R*_s_ value)

The one-pot double derivatization reaction using PTAD and acetylation resulted in separation of some of the product peaks (*R*_*s*_ ˃ 2.0), as shown in Fig. 4. Even though not all product peaks were fully resolved, quantitative analysis can be readily performed using one of the well-resolved product peaks. Similarly, the PyrNO derivatization reaction also successfully separated the epimers (Fig. 5). Finally, FMP derivatives of the epimers showed clear potential for full separation, but would require additional optimization of the chromatographic conditions for the PFP column.

## Conclusions

Chemical derivatization of vitamin D compounds has received significant attention in recent years, with a large selection of different reagents available, some of which are commercially obtainable, while others are custom-made. In this study, we compared 7 different commercially available derivatization reagents for LC–ESI–MS/MS analysis of vitamin D compounds, with respect to improved detection sensitivity and effects on the chromatographic separation. Other aspects concerned the cost of the reagents, the required time for the derivatization step, and the general complexity of the procedure.

In this experimental comparison, differences of stationary and mobile phases, additives, and ion suppression effects potentially affected the measured analyte signals. While all investigated reagents provided significant sensitivity improvements over the analysis of underivatized vitamin D metabolites, Amplifex and FMP-TS yielded the highest gains of detection sensitivity of up to 349-fold, due to the permanently charged moiety of the reagents. The investigated one-pot double derivatization scheme yielded only 2–eightfold sensitivity increases, but offered the advantage of highly selective 25(OH)D_3_ epimer separation.

In terms of chromatographic selectivity, the most complex spectrum of products was generated by PyrNO, due to the formation of four different products and thus four different chromatographic peaks for every analyte. In general, all the reagents’ derivatization products of the dihydroxylated vitamin D_3_ species were more than adequately separated using the experimental chromatographic conditions studied here (*R*_s_ ˃ 2). This was not always the case for the 25(OH)D_3_ epimers. Excellent candidates for the epimer separation were PTAD + Ac double derivatization (*R*_s_ ≥ 2.9), PyrNO (*R*_s_ ≥ 1.9), or INC (*R*_s_ ≥ 1.5).

Importantly, the derivatization reactions used here do not require specialized equipment and can be readily performed in routine analytical laboratories. Most of them were conducted at room temperature, except for FMP-TS (40 °C) and PyrNO (70 °C). The fastest reaction (INC) took only 10 s, while the most time-consuming reaction (PTAD-Ac) needed 2 h to complete. While some of the reagents are expensive (e.g., Amplifex), most of them are relatively inexpensive in comparison to the cost of the other assay components.

In conclusion, unfortunately, there is not a single reagent offering equal enhancement of the detection sensitivity for every vitamin D metabolite, adequate epimer and isomer separation, and low-cost and rapid analysis at the same time. Therefore, we believe that this study can serve as a useful reference for vitamin D laboratories, to help analytical and clinical scientists decide which derivatization reagent to choose for their application. Full validations of the multiple assays compared in this study with respect to linear dynamic range, reproducibility, precision, LOD, and LOQ were beyond the scope of the present investigation, but will be required for the assay using the chosen derivatization reagent.

## Supplementary Information

Below is the link to the electronic supplementary material.

Supplementary file1 (DOCX 40 KB)
