# Supplementary material for: Comparing derivatization reagents for quantitative LC–MS/MS analysis of a variety of vitamin D metabolites
Source: Anal Bioanal Chem. 2023 May 23;415(19):4689–701. doi: 10.1007/s00216-023-04753-0 (PMC10352403; doi:10.1007/s00216-023-04753-0)
Supplement: Supplementary file 1 — Supplementary file1 (DOCX 40 KB) [file 216_2023_4753_MOESM1_ESM.docx]

*Supplementary Information*

**Comparing derivatization reagents for quantitative LC-MS/MS analysis of a variety of vitamin D metabolites**

**Anastasia Alexandridou, Pascal Schorr, Dietrich A. Volmer***

*Bioanalytical Chemistry, Department of Chemistry Humboldt University Berlin, Brook-Taylor-Str. 2, 12489 Berlin, Germany*

*Corresponding author:

Prof. Dr. Dietrich Volmer

Humboldt University Berlin

Department of Chemistry

12489 Berlin, Germany

Tel +49 30 2093 7588

Email: [Dietrich.Volmer@hu-berlin.de](mailto:Dietrich.Volmer@hu-berlin.de)

**Table S1.** Optimized MS/MS (MRM) settings for the vitamin D metabolites and their derivatives.

| **Compound** | **Q1 (*m/z*)** | **Q3 (*m/z*)** | **DP (V)** | **EP (V)** | **CE (V)** | **CXP (V)** |
| --- | --- | --- | --- | --- | --- | --- |
| IS: 25(OH)D_3_-d6 | 407 | 389 | 40 | 11 | 12 | 15 |
|  | 407 | 371 | 40 | 11 | 14 | 15 |
| 3β-/3α-25(OH)D_3_ | 401 | 383 | 40 | 11 | 12 | 15 |
|  | 401 | 365 | 40 | 11 | 14 | 15 |
|  | 401 | 159 | 40 | 11 | 30 | 17 |
| 24,25(OH)_2_D_3_ | 417 | 399 | 50 | 11 | 12 | 14 |
|  | 417 | 381 | 50 | 11 | 12 | 12 |
|  | 417 | 363 | 50 | 11 | 15 | 12 |
| 1,25(OH)_2_D_3_ | 417 | 399 | 150 | 11 | 9 | 13 |
|  | 417 | 381 | 150 | 11 | 13 | 12 |
|  | 417 | 363 | 150 | 11 | 14 | 14 |
| D_3_ | 385 | 367 | 115 | 13 | 15 | 15 |
|  | 385 | 259 | 115 | 13 | 16 | 9 |
|  | 385 | 159 | 115 | 13 | 31 | 19 |
| 3β-/3α-25(OH)D_3_-PTAD | 558 | 298 | 155 | 8 | 21 | 9 |
|  | 558 | 280 | 155 | 8 | 38 | 10 |
|  | 558 | 161 | 155 | 8 | 50 | 19 |
| 24,25(OH)_2_D_3_-PTAD | 592 | 298 | 70 | 12 | 24 | 9 |
|  | 592 | 280 | 70 | 12 | 40 | 13 |
|  | 592 | 161 | 70 | 12 | 52 | 18 |
| 1,25(OH)_2_D_3_-PTAD | 592 | 314 | 45 | 12 | 23 | 14 |
|  | 592 | 268 | 45 | 12 | 44 | 12 |
|  | 592 | 177 | 45 | 12 | 46 | 9 |
| D_3_-PTAD | 560 | 365 | 85 | 14 | 26 | 12 |
|  | 560 | 298 | 85 | 14 | 26 | 13 |
|  | 560 | 280 | 85 | 14 | 50 | 18 |
| 3β-/3α-25(OH)D_3_-PTAD+Ac | 600 | 340 | 125 | 12 | 18 | 10 |
|  | 600 | 280 | 125 | 12 | 32 | 13 |
|  | 600 | 161 | 125 | 12 | 50 | 18 |
| 24,25(OH)_2_D_3_-PTAD+Ac | 676 | 340 | 45 | 11 | 24 | 10 |
|  | 676 | 280 | 45 | 11 | 37 | 13 |
|  | 676 | 161 | 45 | 11 | 59 | 19 |
| 1,25(OH)_2_D_3_-PTAD+Ac | 676 | 598 | 45 | 10 | 16 | 23 |
|  | 676 | 658 | 45 | 10 | 9 | 24 |
|  | 676 | 398 | 45 | 10 | 21 | 14 |
| D_3_-PTAD+Ac | 602 | 542 | 200 | 12 | 21 | 18 |
|  | 602 | 340 | 200 | 12 | 19 | 14 |
|  | 602 | 280 | 200 | 12 | 35 | 13 |
| 3β-/3α-25(OH)D_3_-Amplifex | 732 | 673 | 110 | 11 | 43 | 22 |
|  | 732 | 275 | 110 | 11 | 66 | 14 |
|  | 732 | 217 | 110 | 11 | 69 | 20 |
| 24,25(OH)_2_D_3_-Amplifex / 1,25(OH)_2_D_3_-Amplifex | 748 | 689 | 120 | 11 | 40 | 22 |
|  | 748 | 275 | 120 | 11 | 70 | 13 |
|  | 748 | 217 | 120 | 11 | 70 | 10 |
| D_3_-Amplifex | 716 | 657 | 85 | 12 | 43 | 21 |
|  | 716 | 275 | 85 | 12 | 68 | 13 |
|  | 716 | 217 | 85 | 12 | 68 | 19 |
| 3β-/3α-25(OH)D_3_-PyrNO | 509 | 491 | 120 | 11 | 28 | 16 |
|  | 509 | 473 | 120 | 11 | 30 | 16 |
|  | 509 | 231 | 120 | 11 | 29 | 11 |
| 24,25(OH)_2_D_3_-PyrNO / 1,25(OH)_2_D_3_-PyrNO | 525 | 507 | 120 | 11 | 27 | 16 |
|  | 525 | 489 | 120 | 11 | 30 | 19 |
| 24,25(OH)_2_D_3_-PyrNO | 525 | 231 | 120 | 11 | 30 | 11 |
| 1,25(OH)_2_D_3_-PyrNO | 525 | 247 | 120 | 12 | 29 | 11 |
| D_3_-PyrNO | 493 | 475 | 130 | 12 | 26 | 15 |
|  | 493 | 231 | 130 | 12 | 28 | 10 |
| 3β-/3α-25(OH)D_3_-DMEQ-TAD | 746 | 468 | 80 | 10.5 | 38 | 15 |
|  | 746 | 247 | 80 | 10.5 | 53 | 11.5 |
|  | 746 | 203 | 80 | 10.5 | 120 | 24 |
| 24,25(OH)_2_D_3_-DMEQ-TAD / 1,25(OH)_2_D_3_-DMEQ-TAD | 762 | 468 | 120 | 10.5 | 38 | 15 |
|  | 762 | 247 | 120 | 10.5 | 54 | 11.5 |
|  | 762 | 203 | 120 | 10.5 | 125 | 23 |
| D_3_-DMEQ-TAD | 730 | 468 | 150 | 10.5 | 34 | 15 |
|  | 730 | 247 | 150 | 10.5 | 50 | 28 |
|  | 730 | 203 | 150 | 10.5 | 115 | 23.5 |
| 3β-/3α-25(OH)D_3_-FMP | 492 | 383 | 35 | 13 | 12 | 13 |
|  | 492 | 365 | 35 | 13 | 18 | 13 |
|  | 492 | 159 | 35 | 13 | 35 | 17 |
| 24,25(OH)_2_D_3_- FMP / 1,25(OH)_2_D_3_- FMP | 508 | 399 | 35 | 13 | 13 | 15 |
|  | 508 | 381 | 35 | 13 | 18 | 16 |
|  | 508 | 363 | 35 | 13 | 20 | 13 |
| D_3_-FMP | 476 | 367 | 30 | 13 | 14 | 12 |
|  | 476 | 159 | 30 | 13 | 37 | 18 |
|  | 476 | 145 | 30 | 13 | 40 | 17 |
| 3β-/3α-25(OH)D_3_-INC | 506 | 488 | 110 | 10 | 21 | 16 |
|  | 506 | 365 | 110 | 10 | 25 | 16 |
|  | 506 | 124 | 110 | 10 | 32 | 15 |
| 24,25(OH)_2_D_3_-INC / 1,25(OH)_2_D_3_-INC | 522 | 504 | 95 | 11 | 23 | 17 |
|  | 522 | 124 | 95 | 11 | 33 | 15 |
| 24,25(OH)_2_D_3_-INC | 522 | 486 | 110 | 12 | 27 | 15 |
| 1,25(OH)_2_D_3_-INC | 522 | 381 | 85 | 10 | 25 | 12 |
| D_3_-INC | 490 | 124 | 200 | 10 | 28 | 14 |
|  | 490 | 367 | 200 | 10 | 21 | 16 |

DP: declustering potential, EP: entrance potential, CE: collision energy, CXP: collision cell exit potential

**Scheme S1.** Precursor ions and CID products of the derivatized vitamin D compounds

For acidified solutions, the expected precursor ions for vitamin D compounds are protonated molecules [M+H]^+^, as well as dissociation products after loss of one or two H_2_O molecules in the source, leading to [M+H -H_2_O]^+^ or [M+H -2×H_2_O]^+^. Specifically, for non-derivatized 3α and 3β-25(OH)D_3_ both [M+H]^+^ at *m/z* 401 and [M+H -H_2_O]^+^ at *m/z* 383 were observed. [M+H]^+^ was chosen for the comparison in this study. For 24,25(OH)_2_D_3_ and 1,25(OH)_2_D_3_, one major water loss was observed, leading to at *m/z* 399 ( [M+H]^+^ at *m/z* 417 was also chosen precursor ion). For vitamin D_3_, only [M+H]^+^ at *m/z* 385 was observed.

PTAD attacks the diene moiety of vitamin D compounds, leaving -OH groups intact. Consequently, H_2_O lose is also seen in the CID spectra of the derivatized products. The mass of PTAD-derivatized molecules increases by 175.1 Da. [M_PTAD_+H]^+^ was observed for all analytes; for 3α and 3β-25(OH)D_3_ at *m/z* 576, for 24,25(OH)_2_D_3_ and 1,25(OH)_2_D_3_ at *m/z* 592 for vitamin D_3_ and at *m/z* 560. [M_PTAD_+H-H_2_O]^+^ was observed at *m/z* 558 for 3α and 3β-25(OH)D_3_ and at *m/z* 574 for 24,25(OH)_2_D_3_ and 1,25(OH)_2_D_3_. Finally, [M_PTAD_+H]^+^ was the precursor ion for 24,25(OH)_2_D_3_, 1,25(OH)_2_D_3_ and vitamin D_3_. For 25(OH)D_3_ epimers, [M_PTAD_+H-H_2_O]^+^ was for MRM.

A subsequent derivatization step, following PTAD, was also investigated; namely, acetylation of the hydroxyl groups using acetic anhydride in the presence of pyridine. One or more acetyl groups were expected to react with the molecule, depending on the number of hydroxyl groups, leading to multiple precursor ions. For 25(OH)D_3_ epimers, (1) [M_PTAD+Ac_+H]^+^ and (2) [M_PTAD+Ac_+H -H_2_O]^+^ or [M_PTAD+2×Ac_+H -CH_3_COOH]^+^ were observed as precursor ions; at *m/z* 618 and 600 for 3α-25(OH)D_3_ and 3β-25(OH)D_3_. Vitamin D_3_ formed only [M_PTAD+Ac_+H]^+^ at *m/z* 602, without possibility for subsequent H_2_O loss in the source. The dihydroxylated species led to a more complex scenario because of several combinations of adducts and fragmentations in the source. Two precursor ions were observed for 24,25(OH)_2_D_3_; *m/z* 676 (corresponding to [M_PTAD+2×Ac_+H]^+^) and *m/z* 658 (corresponding to [M_PTAD+2×Ac_+H-H_2_O]^+^ or [M_PTAD+3×Ac_+H-CH_3_COOH]^+^). Four precursor ions were seen for 1,25(OH)_2_D_3_; 1); at *m/z* 676 for [M_PTAD+2×Ac_+H]^+^) at *m/z* 658 for [M_PTAD+2×Ac_+H-H_2_O]^+^ or [M_PTAD+3×Ac_+H -CH_3_COOH]^+^, at *m/z* 616 for [M_PTAD+Ac_+H-H_2_O]^+^ or [M_PTAD+2×Ac_+H -CH_3_COOH]^+^ and at *m/z* 598 for [M_PTAD+Ac_+H-2×H_2_O]^+^ or [M_PTAD+3×Ac_+H-2×CH_3_COOH]^+^. Finally, [M_PTAD+Ac_+H-H_2_O]^+^ was chosen for 3α and 3β-25(OH)D_3_, [M_PTAD+Ac_+H]^+^ for vitamin D_3_ and [M_PTAD+2×Ac_+H]^+^ for 24,25(OH)_2_D_3_ and 1,25(OH)_2_D_3_.

Amplifex is a dienophilic reagent similar to PTAD, exhibiting a permanently-charged quaternary group. [M_Amplifex_]^+^ was the major precursor ion for all derivatization products; at *m/z* 732 for 3α and 3β-25(OH)D_3_, at *m/z* 748 for 24,25(OH)_2_D_3_ and 1,25(OH)_2_D_3_, and at *m/z* 716 for vitamin D_3_. The quaternary ammonium group was the common leaving group upon activation at *m/z* 59 for all investigated derivatization products.

FMP-TS generally reacts with alcohols, leading to a permanently charged-pyridinium moiety via nucleophilic substitution. For vitamin D_3_ species, all non-tertiary hydroxyl groups can potentially react. The precursor ion for derivatized vitamin D_3_ was seen at *m/z* 476. For 3α and 3β-25(OH)D_3_, reaction with only a single hydroxyl group was observed, leading *m/z* 492. Three precursor ions were observed for 24,25(OH)_2_D_3_: [M_FMP-TS_]^+^ at *m/z* 508, [M_FMP-TS_-H_2_O]^+^ at *m/z* 490 and [M_2×FMP-TS_]^2+^ at *m/z* 300. Two precursor ions were observed for 1,25(OH)_2_D_3_: [M_FMP-TS_]^+^ at *m/z* 508 and [M_FMP-TS_-H_2_O]^+^ at *m/z* 490. For 24,25(OH)_2_D_3_ and 1,25(OH)_2_D_3_, the *m/z* 508 was selected precursor ion, since it exhibited the highest abundance.

As an acyl chloride, INC performs an acylation reaction with alcohols to give esters: [M_INC_+H]^+^ at *m/z* 506, [M_2×INC_+H]^+^ at *m/z* 611 and [M_2×INC_+H]^2+^ at *m/z* 306 as precursor ions for 3α and 3β-25(OH)D_3_. Similarly, [M_INC_+H]^+^ at *m/z* 522, [M_2×INC_+H]^+^ at *m/z* 627 and [M_3×INC_+H]^+^ at *m/z* 732 for 24,25(OH)_2_D_3_ and 1,25(OH)_2_D_3_. As expected, [M_INC_+H]^+^ at *m/z* 490 was the only precursor ion for vitamin D_3_. [M_INC_+H]^+^ was chosen for further fragmentation for all analytes.

PyrNO is a dienophile for hetero-Diels-Alder reaction with vitamin D_3_. [M_PyrNO_+H]^+^ was precursor ion for all analytes; at *m/z* 509 for 3α-25(OH)D_3_ and 3β-25(OH)D_3_, at *m/z* 525 for 24,25(OH)_2_D_3_ and 1,25(OH)_2_D_3_, and at *m/z* 493 for vitamin D_3_. For all species except for vitamin D_3_, [M_PyrNO_+H-H_2_O]^+^ ions were observed at much lower abundanc than [M_PyrNO_+H]^+^.

DMEQ-TAD is a Cookson-type reagent, similar to PTAD and Amplifex. [M_DMEQ-TAD_+H]^+^ was the precursor ion for all analytes: at *m/z* 746 for 3α-25(OH)D_3_ and 3β-25(OH)D_3_, at *m/z* 762 for 24,25(OH)_2_D_3_ and 1,25(OH)_2_D_3_, and at *m/z* 730 for vitamin D_3_. For 3α-25(OH)D_3_ and 3β-25(OH)D_3_, [M_DMEQ-TAD_+H-H_2_O]^+^ was also observed at *m/z* 728.

**Table S2.** Retention times of five vitamin D_3_ metabolites, their derivatization products and IS (25(OH)D_3_-d6) under different chromatographic conditions.*

| **Retention Time (min)** | ***Chromatographic conditions (Column/Mobile phase)*** | | | |
| --- | --- | --- | --- | --- |
| ***Compound*** | ***C-18/H_2_O-MeOH*** | ***C-18/H_2_O-ACN*** | ***F5/H_2_O-MeOH*** | ***F5/H_2_O-ACN*** |
| D_3_ | 14.64 | 14.58 | 12.50 | 7.71 |
| 3β-25(OH)D_3_ | 11.42 | 6.97 | 9.96 | 3.24 |
| 1,25(OH)_2_D_3_ | 9.83 | 3.37 | 8.57 | 1.78 |
| 3α-25(OH)D_3_ | 11.44 | 6.99 | 10.14 | 3.32 |
| 24,25(OH)_2_D_3_ | 9.23 | 3.51 | 7.93 | 1.76 |
| 25(OH)D_3_-d6 | 11.37 | 6.88 | 9.94 | 3.20 |
| D_3_-PTAD | 12.90 | 10.26/10.11 | 11.32/11.22 (splitting) | 5.38/5.24 |
| 3β-25(OH)D_3_-PTAD | 9.32/9.14 | 3.69/3.50 | 8.52/8.34 | 2.02/1.92 (splitting) |
| 1,25(OH)_2_D_3_-PTAD | 8.16 | 2.02/2.08 (splitting) | 7.33 | 1.30 |
| 3α-25(OH)D_3_-PTAD | 9.24 | 3.56/3.70 | 8.47/8.40 (splitting) | 2.01/1.94 (splitting) |
| 24,25(OH)_2_D_3_-PTAD | 7.35/6.59 | 1.82/1.61 | 6.77/6.14 | 1.24/1.14 (splitting) |
| D_3_-PTAD+Ac | 13.84/13.60 | 12.99/12.27 | 12.48/12.35 | 7.33/7.14 |
| 3β-25(OH)D_3_-PTAD+Ac | 10.57/10.40/  10.91/11.10 | 6.36/5.92/  7.75/8.16 | 10.06/11.05/  11.16 | 3.34/4.93 (splitting) |
| 1,25(OH)_2_D_3_-PTAD+Ac | 10.38/9.89 | 6.08/5.44 | 10.27/9.61 | 3.37/3.07 |
| 3α-25(OH)D_3_-PTAD+Ac | 10.04/10.62/  10.76/11.26 | 5.55/6.42/  7.40/8.22 | 9.80/10.17/  10.94/11.22 | 3.10/3.43/  4.66/5.01 |
| 24,25(OH)_2_D_3_-PTAD+Ac | 9.56 (splitting) | 4.96/4.75 | 9.50 | 2.84 |
| D_3_-Amplifex | 9.92/10.13 | 4.21/4.44 | 10.34 | 3.43 (splitting) |
| 3β-25(OH)D_3_- Amplifex | 6.61 | 1.11 | 7.36/7.24 (splitting) | 1.15 |
| 1,25(OH)_2_D_3_- Amplifex | 5.54/5.64 (splitting) | 0.77 | 6.21/6.09 (splitting) | 0.86 |
| 3α-25(OH)D_3_- Amplifex | 6.54/6.70 (splitting) | 1.14 (splitting) | 7.39/7.24 (splitting) | 1.14 |
| 24,25(OH)_2_D_3_- Amplifex | 4.70/4.26 | 0.71 | 5.50/5.10 | 0.81 |
| D_3_-FMP | 11.40 | 8.05 | 12.25 | 7.49 |
| 3β-25(OH)D_3_- FMP | 8.14 | 2.77 | 8.97 | 2.42 |
| 1,25(OH)_2_D_3_- FMP | 6.96 | 1.44 | 7.54 | 1.47 |
| 3α-25(OH)D_3_- FMP | 8.25 | 2.83 | 9.16 | 2.56 |
| 24,25(OH)_2_D_3_- FMP | 5.85 | 1.18 | 6.72 | 1.36 |
| D_3_-INC | 15.82 | Not detected | 14.08 | 10.56 |
| 3β-25(OH)D_3_- INC | 13.38 | 11.65 | 12.23 | 5.84 |
| 1,25(OH)_2_D_3_- INC | 10.68/11.57 | 5.16/6.58 | 9.90/10.70 | 2.61/3.37 |
| 3α-25(OH)D_3_- INC | 13.39 | 11.75 | 12.45 | 6.02 |
| 24,25(OH)_2_D_3_- INC | 11.86 | 7.64 | 10.86 | 3.65 |
| D_3_-PyrNO | 12.54/12.03 & 13.52/13.21 | 8.20/6.80 & 10.53/9.57 | 10.82/10.34 & 11.86/11.44 | 3.83/3.46 & 4.85/4.33 |
| 3β-25(OH)D_3_- PyrNO | 7.90/7.14 & 9.85/9.09 | 1.70/1.36 & 2.77/2.20 | 7.01/6.31 & 8.78/7.97 | 1.16/1.06 & 1.50/1.31 |
| 1,25(OH)_2_D_3_- PyrNO | 6.06/6.43 & 8.12/8.35 | 0.94/0.84 & 1.22/1.44 | 5.62/5.19 & 6.95/7.43 | 0.82 & 0.95/1.02 (splitting) |
| 3α-25(OH)D_3_- PyrNO | 6.99/8.12 & 9.40/9.60 | 1.34/1.75 & 2.47 | 6.21/7.08 & 8.47/8.32 | 1.07/1.13 (splitting) & 1.40 |
| 24,25(OH)_2_D_3_- PyrNO | 5.14/3.79 & 7.62/5.91 | 0.84/0.73 & 1.25/0.95 | 4.50/3.46 & 6.64/5.16 | 0.79 & 0.94/0.82 |
| D_3_-DMEQ-TAD | 12.40/12.07 | 8.24/7.69 | 11.64/11.14 | 4.29/3.92 |
| 3β-25(OH)D_3_- DMEQ-TAD | 8.51/8.04 | 2.36/2.06 | 8.70/8.06 | 1.48/1.33 |
| 1,25(OH)_2_D_3_- DMEQ-TAD | 6.96/7.52 | 1.21/1.36 | 7.03/7.62 | 0.97 (splitting) |
| 3α-25(OH)D_3_- DMEQ-TAD | 8.52/8.04 | 2.38/2.06 | 8.57/8.13 | 1.43/1.36 |
| 24,25(OH)_2_D_3_- DMEQ-TAD | 6.43/5.54 | 1.18/0.98 | 6.94/5.88 | 0.96/0.86 |

*In cases where two retention times are given, these correspond to two different peaks of the same analyte, with the first number referring to the largest peak.
